# Correlation of vaginal lactic acid bacteria changes with high-risk human papillomavirus-infected cervical intraepithelial neoplasia and nomogram model

**DOI:** 10.3389/fmed.2026.1784053

**Published:** 2026-03-20

**Authors:** Nianxue Chen, Lingna Sun

**Affiliations:** Key Laboratory of Maternal and Fetal Medicine of National Health Commission of China, Department of Gynecology, Shandong Provincial Maternal and Child Health Care Hospital Affiliated to Qingdao University, Jinan, China

**Keywords:** cervical intraepithelial neoplasia, HR-HPV infection, nomogram, risk, vaginal lactobacilli

## Abstract

**Objective:**

To investigate the correlation between vaginal lactobacilli and intraepithelial neoplasia (CIN) and high-risk infection of human papillomavirus (HR-HPV).

**Methods:**

A total of 358 HR-HPV-infected women who underwent gynecological examinations were selected as the study subjects. The HR-HPV-infected women were randomly divided into a training set (*n* = 251) and a validation set (*n* = 107) at a ratio of 7:3. In the training set, influencing factors associated with the occurrence of CIN were screened by multivariate Logistic regression analysis, and a Nomogram prediction model was constructed. The predictive performance of the model was evaluated by plotting the receiver operating characteristic (ROC) curve and calibration curve, and then validated in the validation set. Meanwhile, decision curve analysis (DCA) was used to evaluate the clinical application value of the Nomogram model.

**Results:**

The incidence of CIN was 54.98% (138/251) in the training set and 53.27% (57/107) in the validation set, with no significant differences in the incidence of CIN and clinical characteristics between the two groups (*P* > 0.05). In the training set, a decrease in the number of vaginal lactobacilli, an increase in vaginal pH, a higher HR-HPV load, an older age, a longer duration of persistent HPV infection, and a decrease in serum vitamin D level were independent risk factors for the occurrence of CIN in HR-HPV-infected women (all *P* < 0.05), and a Nomogram prediction model was further constructed. The Nomogram model showed good calibration and goodness-of-fit in both the training set and the validation set (C-index values were 0.845 and 0.761, and the *P*-values of the Hosmer–Lemeshow test were 0.368 and 0.412, respectively). The ROC curves showed that the areas under the curve (AUCs) of the Nomogram model for predicting the occurrence of CIN in HR-HPV-infected women in the training set and the validation set were 0.845 (95% CI: 0.767–0.923) and 0.761 (95% CI: 0.611–0.910), respectively, with sensitivities and specificities of 0.660, 0.845, and 0.619, 0.725, respectively.

**Conclusion:**

The Nomogram is helpful for early prediction of CIN in HR-HPV-infected women and guiding the formulation of appropriate clinical intervention measures.

## Introduction

Cervical intraepithelial neoplasia (CIN) is a crucial stage of cervical precancerous lesions, and its occurrence is closely associated with persistent infection with high-risk human papillomavirus (HR-HPV) (1). Although most HR-HPV infections can be cleared spontaneously, 10–13% of infected individuals still progress to CIN, seriously threatening female reproductive health (2). Imbalance of the vaginal microecology, especially the decrease in the number of lactobacilli, has been confirmed to be related to HR-HPV infection and lesion progression. Meanwhile, the regulatory effects of factors such as serum vitamin D level and viral load have also gradually attracted attention (3, 4). Currently, there was a lack of tools for accurately assessing the risk of CIN occurrence in HR-HPV infected individuals in clinical practice, resulting in a lack of individualized and targeted intervention measures. Based on clinical practice and previous pre-test data, this study included 358 patients with HR-HPV infection. By screening age, vaginal microecology, virological and serological related indicators, a nomogram model for predicting the risk of CIN occurrence was constructed and validated. The independent effects and weights of each factor were clarified to provide a scientific basis for early risk stratification and the formulation of precise intervention strategies, ultimately reducing the incidence of precancerous cervical lesions and cervical cancer.

## Materials and methods

### Study population

Patients diagnosed with HR-HPV infection in the gynecological outpatient department and inpatient department of our hospital from January 2021 to June 2024 were included. The patients were randomly divided into a training set and a validation set at a ratio of 7:3.

Inclusion criteria were as follows: Age > 18 years; Pathologically diagnosed with cervical intraepithelial neoplasia (CIN); HR-HPV infection confirmed by PCR, next-generation sequencing (NGS), or hybrid capture method (such as HC2); complete clinical data (including TCT, HPV typing, vaginal microecology test, pathological report, and follow-up data); voluntary participation in this study and signing of the informed consent form. Exclusion criteria were as follows: comorbid with other infections (such as bacterial vaginitis, trichomoniasis, or vulvovaginal candidiasis); received antibiotic, vaginal suppository, or immunomodulatory treatment within 3 months; pregnant or lactating; suffering from immunodeficiency diseases (such as HIV infection, long-term use of immunosuppressants); comorbid with cervical cancer or other gynecological malignancies; suffering from autoimmune diseases (such as systemic lupus erythematosus, rheumatoid arthritis).

### Data collection

The following information was collected through the hospital’s electronic medical record system, gynecological examination database, and patient follow-up management system.

Basic patient information and clinical characteristics: demographic data: age, marital status (unmarried/married/divorced/widowed). Lifestyle habits and past medical history: smoking history (yes/no), drinking history (yes/no), past history of gynecological inflammation (vaginitis/cervicitis/others/no).

Reproductive health-related characteristics: number of pregnancies and deliveries, menstrual cycle, menstrual duration, menstrual volume (low/normal/high), age at menarche, number of sexual partners, age at first sexual intercourse, contraceptive method (condom/oral contraceptive/intrauterine device/others).

Vaginal microecology and etiological assessment parameters (baseline measurement before testing): vaginal microecology indicators: types of lactobacilli (Lactobacillus crispatus/Lactobacillus gasseri/Lactobacillus jensenii), relative abundance of lactobacilli, vaginal pH value, Nugent score, vaginal flora diversity index (Shannon index).

HR-HPV-related tests: HR-HPV typing (HPV16/HPV18/ HPV31/HPV33, etc.), HR-HPV viral load, duration of persistent HPV infection.

Pathological and inflammatory factor tests: pathological biopsy results (negative/low-grade lesion/high-grade lesion), interleukin-6 (Interleukin-6, IL-6) level, interleukin-10 (Interleukin-10, IL-10) level, serum vitamin D level.

### Outcome definition

Referring to the “Guidelines for Cervical Cancer Screening and Prevention (2023 Edition)” (5) and the international diagnostic and grading standards for cervical intraepithelial neoplasia (WHO Classification of Tumors of the Female Genital Organs, 2020), and considering the characteristics of the follow-up cycle of the HR-HPV infected population in this study, based on cervical histopathological examination and clinical test results, the patients’ outcomes were divided into two groups.

CIN occurrence group: At the 12-month follow-up, meeting any of the following conditions: (1) histopathological diagnostic criteria: diagnosed with cervical intraepithelial neoplasia (CIN) grade I or above (CIN I, CIN II, CIN III) by cervical biopsy under colposcopy or pathological examination after cervical conization. The pathological report clearly showed atypical hyperplasia of cervical epithelial cells, and the scope of the lesion involving the epithelial layer met the corresponding grading criteria; (2) clinical intervention indications: due to the pathological results indicating a risk of lesion progression (such as CIN II or above), the patient received targeted intervention treatments such as cervical conization or laser treatment; (3) lesion progression during follow-up: No CIN at baseline, and new CIN lesions were confirmed by repeated pathological examinations during the follow-up (6 months, 12 months), excluding interference from tumor metastasis from other sites or benign cervical lesions (such as inflammation, polyps) (6).

CIN non-occurrence group: at the 12-month follow-up, meeting all of the following conditions: (1) negative histopathology: cervical cytology examination (TCT) was normal at each follow-up time point (6 months, 12 months), and the pathological report of biopsy under colposcopy showed no atypical hyperplasia of cervical epithelial cells, only normal cervical tissue or chronic cervicitis (without cytological atypia); (2) no lesion-related symptoms and interventions: the patient had no symptoms related to cervical lesions such as contact bleeding or irregular vaginal bleeding, and did not receive any invasive intervention treatment for cervical lesions; (3) follow-up stability: the HR-HPV infection status could fluctuate (turn negative or persistent infection) during the follow-up, but there was always no pathological evidence of CIN, and the pathological result at the last follow-up (12 months) was still negative (7). All outcome assessments were conducted by gynecologists or pathologists who had received unified training at three time points: at enrollment (baseline), 6-month follow-up, and 12-month follow-up. Histopathological assessment was completed through cervical cytology examination (TCT), high-risk HPV testing, and biopsy under colposcopy. Difficult cases were double-blindly reviewed by two senior pathologists. Clinical symptom assessment was collected through a structured questionnaire, and relevant symptoms such as abnormal vaginal discharge and contact bleeding were recorded simultaneously. Outcome determination (grouping into occurrence or non-occurrence) was independently completed by two researchers who were unaware of the patients’ baseline lactobacilli and vitamin D levels. When there was a disagreement, the third chief physician of the project team made an arbitration based on the pathological report, clinical symptoms, and follow-up records. All assessment data were entered into an electronic data collection system (EDC), and rules for pathological diagnosis coding verification and symptom logic verification were set to ensure the accuracy and integrity of the data.

### Sample size calculation

Based on the expected incidence of the primary outcome indicator (occurrence of cervical intraepithelial neoplasia in HR-HPV infected individuals), which was set at 10–13% [consistent with the actual clinical incidence according to the reported incidence of cervical intraepithelial neoplasia in HR-HPV infected populations in previous literature, data from previous small-sample pre-tests, and characteristics of the target population (HR-HPV infected patients)], a protocol matching the core statistical method (multivariate Logistic regression, used to analyze the associations between clinical, microbiological, and serological indicators and the occurrence of cervical intraepithelial neoplasia) was adopted. The calculation was completed using the “module of SPSS 26.0 and verified using the “pwr” and “epiR” packages of R 4.2.1. The significance level was set at α = 0.05 (two-tailed), and the test power was set at 1-β = 80%. Considering possible patient loss to follow-up and unqualified specimens during the study, an additional 15% sample size redundancy was added. Finally, the minimum required sample size was 286 cases.

### Statistical analysis

Data analysis was completed using SPSS 26.0 and R 4.2.3. Normality of quantitative data was assessed using the Shapiro–Wilk test. Normally distributed data are presented as mean ± standard deviation and were compared using the independent-samples t-test, while non-normally distributed data are expressed as median (interquartile range) and were compared with the Mann–Whitney *U* test. Categorical data were expressed as number of cases (percentage) [*n* (%)] (all candidate variables in this study were continuous variables. Comparison between groups was performed using the χ^2^ test. If the theoretical frequency *T* < 5, the Fisher’s exact probability method was used. In the training set, univariate analysis and multivariate logistic regression analysis were used to determine the independent influencing factors for the occurrence of CIN, and the regression coefficient (B), standard error (SE), Wald value, *P*-value, odds ratio (OR), and 95% confidence interval (CI) of each factor were calculated. Based on the independent influencing factors, a nomogram model for predicting the risk of CIN occurrence was constructed. Internal validation of the training set was performed by the Bootstrap method (1,000 repeated samplings) in the validation set. The concordance index (C-index) was used to evaluate the discrimination ability of the model. Calibration curves were drawn, and the calibration degree of the model was evaluated by the degree of fit between the curve and the diagonal. The *P*-value of the goodness-of-fit calibration test was calculated. The receiver operating characteristic (ROC) curve was drawn, and the area under the curve (AUC) and its 95% CI were calculated to evaluate the prediction accuracy of the model in the training set and the validation set. The clinical net benefit of the model was verified by decision curve analysis (DCA). Model stability verification followed the principle of “at least 5–10 events per variable (EPV).” In this study, the number of CIN occurrence events and the number of included predictive variables needed to satisfy EPV ≥ 5. Multicollinearity was tested by the variance inflation factor (VIF). If VIF < 2, the collinearity between variables was considered low, and the model stability was good. SHapley Additive exPlanations (SHAP) analysis was performed using the “shapley” package of R software to quantify the relative importance of each predictive variable and its direction of influence on the outcome, enhancing the interpretability of the model. All statistical analyses were set with a significance level of α = 0.05, two-tailed test.

## Results

### Comparison of general information of patients in the training set and the validation set

A total of 358 patients with HR-HPV infection were included in this study and randomly divided into a training set (*n* = 251) and a validation set (*n* = 107). In the training set, there were 138 (54.98%) patients with CIN. In the validation set, there were 57 (53.27%) patients with CIN. There were no statistically significant differences in the CIN incidence and general data between the training set and the validation set (*P* > 0.05), indicating that the data set was evenly divided and had good comparability (Table 1).

**TABLE 1:** Comparison of general information of patients in the training set and the validation set.

Indicators		Training set (*n* = 251)	Validation set (*n* = 107)	*t*/χ^2^	*P*
Age (years)		34.62 ± 7.85	35.18 ± 8.21	0.630	0.529
Marital status (%)	Unmarried	32 (12.75)	14 (13.08)	2.150	0.542
	Married	201 (80.08)	85 (79.44)		
	Divorced	15 (5.98)	6 (5.61)		
	Widowed	3 (1.19)	2 (1.87)		
Smoking history (yes/no)		42 (16.73)/209 (83.27)	18 (16.82)/89 (83.18)	0.002	0.964
Drinking history (yes/no)		58 (23.11)/193 (76.89)	25 (23.36)/82 (76.64)	0.008	0.928
Number of pregnancies and deliveries (times)		2.35 ± 1.12	2.41 ± 1.08	0.470	0.638
Menstrual cycle (days)		28.42 ± 2.36	28.75 ± 2.51	1.150	0.251
Menstrual duration (days)		4.58 ± 1.23	4.72 ± 1.19	0.980	0.328
Menstrual volume (%)	Low	35 (13.94)	16 (14.95)	1.870	0.397
	Normal	198 (78.88)	83 (77.57)		
	High	18 (7.17)	8 (7.48)		
Age at menarche (years)		13.65 ± 1.52	13.81 ± 1.47	0.920	0.358
Number of sexual partners		1.82 ± 0.95	1.75 ± 0.89	0.680	0.497
Age at first sexual intercourse (years)		22.36 ± 2.78	22.61 ± 2.65	0.790	0.431
Contraceptive method (%)	Condom	112 (44.62)	48 (44.86)	3.260	0.353
	Oral contraceptives	35 (13.94)	13 (12.15)		
	Intrauterine device	88 (35.06)	39 (36.44)		
	Others	16 (6.37)	7 (6.54)		
History of previous gynecological inflammation (%)	Vaginitis	65 (25.89)	29 (27.10)	2.530	0.282
	Cervicitis	48 (19.12)	21 (19.63)		
	Others	18 (7.17)	6 (5.61)		
	None	120 (47.81)	51 (47.66)		
Types of lactic acid bacteria (%)	Lactobacillus crispatus	132 (52.59)	56 (52.34)	1.980	0.373
	Lactobacillus gasseri	78 (31.08)	34 (31.78)		
	Lactobacillus jensenii	41 (16.33)	17 (15.89)		
Vaginal pH value		4.28 ± 0.35	4.32 ± 0.32	1.050	0.294
Relative abundance of lactic acid bacteria (%)		68.45 ± 15.72	67.83 ± 16.21	0.380	0.704
Nugent score (points)		3.21 ± 1.85	3.35 ± 1.79	0.690	0.491
Vaginal flora diversity index (Shannon index)		1.85 ± 0.62	1.92 ± 0.58	1.010	0.312
HR-HPV typing (%)	HPV16	98 (39.04)	42 (39.25)	2.760	0.431
	HPV18	56 (22.31)	24 (22.43)		
	HPV31	45 (17.93)	18 (16.82)		
	HPV33	52 (20.72)	23 (21.49)		
HR-HPV viral load ( × 10^4^ copies/mL)		5.82 ± 3.15	6.05 ± 2.98	0.670	0.503
Duration of persistent HPV infection (months)		11.35 ± 4.82	10.98 ± 5.13	0.650	0.516
Pathological biopsy results (%)	Negative	28 (11.16)	13 (12.15)	2.890	0.237
	Low-grade lesion	85 (33.86)	37 (34.58)		
	High-grade lesion	138 (54.98)	57 (53.27)		
IL-6 (pg/mL)		12.85 ± 4.32	13.12 ± 4.15	0.610	0.542
IL-10 (pg/mL)		8.36 ± 2.75	8.58 ± 2.69	0.730	0.465
Serum vitamin D level (ng/mL)		21.45 ± 5.82	20.98 ± 6.13	0.780	0.436

### Univariate analysis of effective influencing factors

In the training set, univariate analysis revealed statistically significant differences in age, vaginal pH value, relative abundance of lactic acid bacteria, HR-HPV viral load, duration of persistent HPV infection, and serum vitamin D level between the CIN and non-CIN groups (all *P* < 0.05) (Table 2).

**TABLE 2:** Univariate analysis of influencing factors of CIN in HR-HPV infected patients.

Indicators		CIN occurrence group (*n* = 138)	CIN non-occurrence group (*n* = 107)	*t*/χ^2^	*P*
Age (years)		38.85 ± 7.52	32.42 ± 6.18	4.432	< 0.001
Marital status (%)	Unmarried	15 (10.87)	17 (15.89)	1.326	0.723
	Married	112 (81.16)	82 (76.64)		
	Divorced	9 (6.52)	6 (5.61)		
	Widowed	2 (1.45)	2 (1.87)		
Smoking history (yes/no)		25(18.12)/113 (81.88)	17 (15.89) / 90 (84.11)	0.218	0.640
Drinking history (yes/no)		32 (23.19)/106 (76.81)	26 (24.30) / 81 (75.70)	0.057	0.811
Number of pregnancies and deliveries (times)		2.58 ± 1.24	2.21 ± 1.03	1.985	0.570
Menstrual cycle (days)		28.65 ± 2.48	28.32 ± 2.27	0.976	0.330
Menstrual duration (days)		4.72 ± 1.31	4.45 ± 1.15	1.423	0.156
Menstrual volume (%)	Low	18 (13.04)	17 (15.89)	0.582	0.747
	Normal	110 (79.71)	83 (77.57)		
	High	10 (7.25)	7 (6.54)		
Age at menarche (years)		13.72 ± 1.58	13.58 ± 1.46	0.643	0.521
Number of sexual partners		2.05 ± 1.02	1.78 ± 0.87	1.892	0.660
Age at first sexual intercourse (years)		22.15 ± 2.85	22.58 ± 2.62		
Contraceptive method (%)	Condom	62 (44.93)	46 (42.99)	1.124	0.262
	Oral contraceptives	19 (13.77)	16 (14.95)		
	Intrauterine device	48 (34.78)	38 (35.51)		
	Others	9 (6.52)	7 (6.54)		
History of previous gynecological inflammation (%)	Vaginitis	35 (25.36)	29 (27.10)	0.287	0.960
	Cervicitis	28 (20.29)	21 (19.63)		
	Others	10 (7.25)	8 (7.48)		
	None	65 (47.10)	49 (45.79)		
Types of lactic acid bacteria (%)	Lactobacillus crispatus	72 (52.17)	58 (54.21)	0.198	0.906
	Lactobacillus gasseri	45 (32.61)	34 (31.78)		
	Lactobacillus jensenii	21 (15.22)	15 (14.02)		
Vaginal pH value		4.55 ± 0.38	4.12 ± 0.25	5.826	< 0.001
Relative abundance of lactic acid bacteria (%)		60.35 ± 14.82	72.61 ± 10.37	4.591	< 0.001
Nugent score (points)		4.12 ± 1.95	4.35 ± 1.72	2.876	0.486
Vaginal flora diversity index (Shannon index)		2.15 ± 0.68	1.82 ± 0.55	3.218	< 0.001
HR-HPV typing (%)	HPV16	55 (39.86)	43 (40.19)	0.105 0.997	0.997
	HPV18	31 (22.46)	24 (22.43)		
	HPV31	25 (18.12)	18 (16.82)		
	HPV33	27 (19.56)	22 (20.56)		
HR-HPV viral load ( × 10^4^ copies/mL)		7.25 ± 3.58	3.12 ± 1.85	5.997	< 0.001
Duration of persistent HPV infection (months)		15.82 ± 5.26	8.02 ± 3.18	7.665	< 0.001
Pathological biopsy results (%)	Negative	12 (8.70)	92 (85.98)	6.254	0.568
	Low-grade lesion	45 (32.61)	13 (12.15)		
	High-grade lesion	81 (58.69)	2 (1.87)		
IL-6 (pg/mL)	7.25 ± 3.58	3.12 ± 1.85	1.624	0.106
IL-10 (pg/mL)	15.82 ± 5.26	8.02 ± 3.18	1.138	0.256
Serum vitamin D level (ng/mL)	17.65 ± 5.32	25.82 ± 6.15	7.524	< 0.001

### Multivariate logistic regression analysis of influencing factors

Taking whether CIN occurred in HR-HPV infected patients as the dependent variable (1 = occurrence group, 0 = non-occurrence group), the indicators with statistical significance in the univariate analysis were included in the multivariate Logistic regression analysis. The results showed that age, vaginal pH value, relative abundance of lactic acid bacteria, HR-HPV viral load, duration of persistent HPV infection, and serum vitamin D level were significantly associated with CIN occurrence (all *P* < 0.05). Age, vaginal pH value, HR-HPV viral load, and duration of persistent HPV infection were independent risk factors, while the relative abundance of lactic acid bacteria and serum vitamin D level were independent protective factors (Table 3).

**TABLE 3:** Multivariate logistic regression analysis of influencing factors of CIN in HR-HPV infected patients.

Factors	β	SE	Wald	*P*	OR	95%CI
Age	0.162	0.069	5.498	0.019	1.175	1.027–1.345
Vaginal pH value	4.086	1.463	7.800	0.005	5.493	3.382–6.474
Relative abundance of lactic acid bacteria	−0.077	0.034	5.086	0.024	0.926	0.866–0.990
HR-HPV viral load	0.548	0.169	10.529	0.001	1.729	1.242–2.407
Duration of persistent HPV infection	0.323	0.106	9.291	0.002	1.382	1.122–1.701
Serum vitamin D level	−0.169	0.066	6.650	0.010	0.844	0.742–0.960
Constant	−18.940	7.576	6.250	0.012		

### Construction of the nomogram prediction model

**FIGURE 1:**
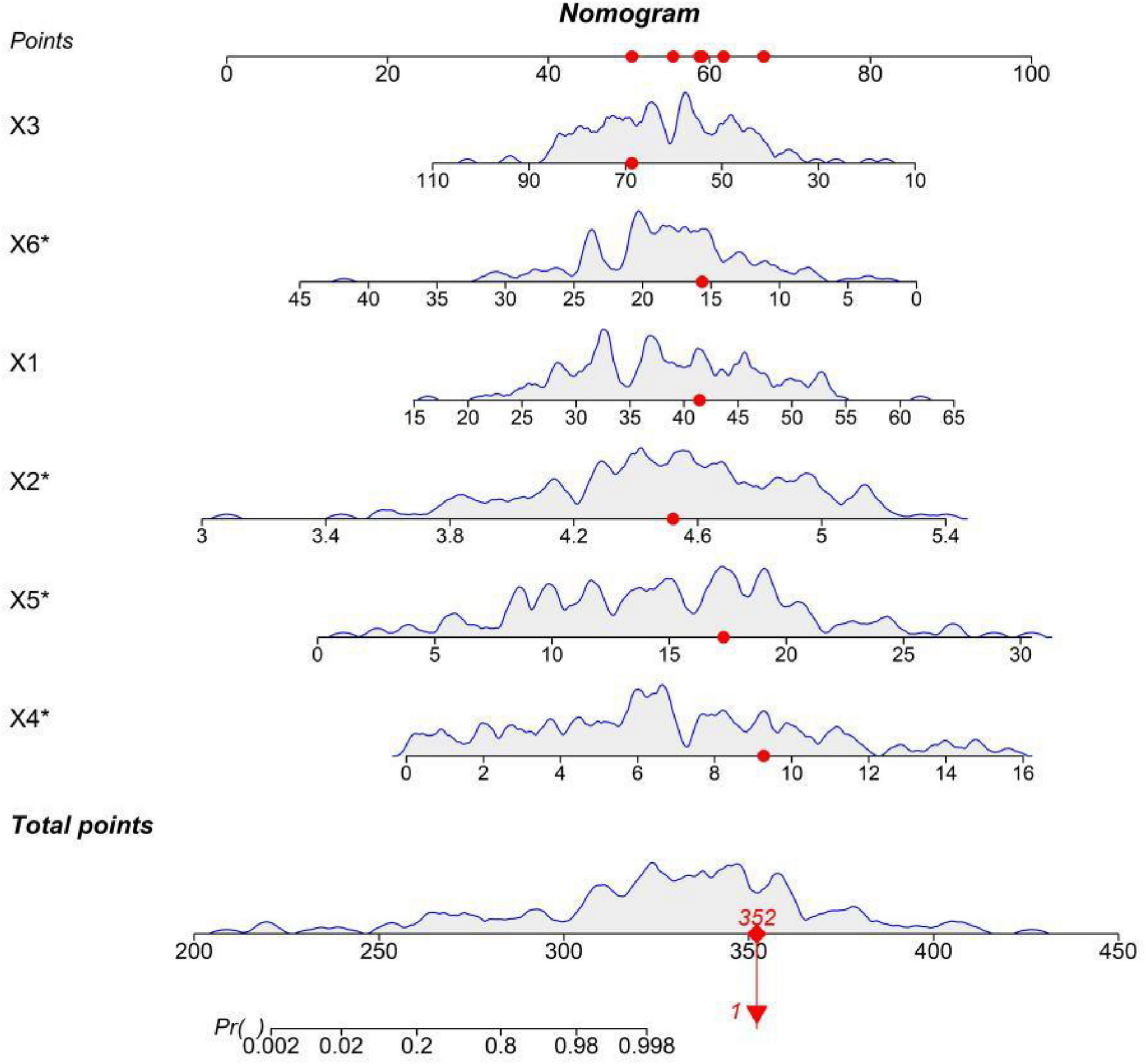
Nomogram for predicting the risk of CIN occurrence (X1, age; X2, vaginal pH value; X3, relative abundance of lactic acid bacteria; X4, HR-HPV viral load; X5, duration of persistent HPV infection; X6, serum vitamin D level). * is a marker for the core predictive indicators of the study, to highlight the four independent risk factors (X2: vaginal pH value, X4: HR-HPV viral load, X5: duration of persistent HPV infection, X6: serum vitamin D level) for constructing the nomogram prediction model.

**FIGURE 2:**
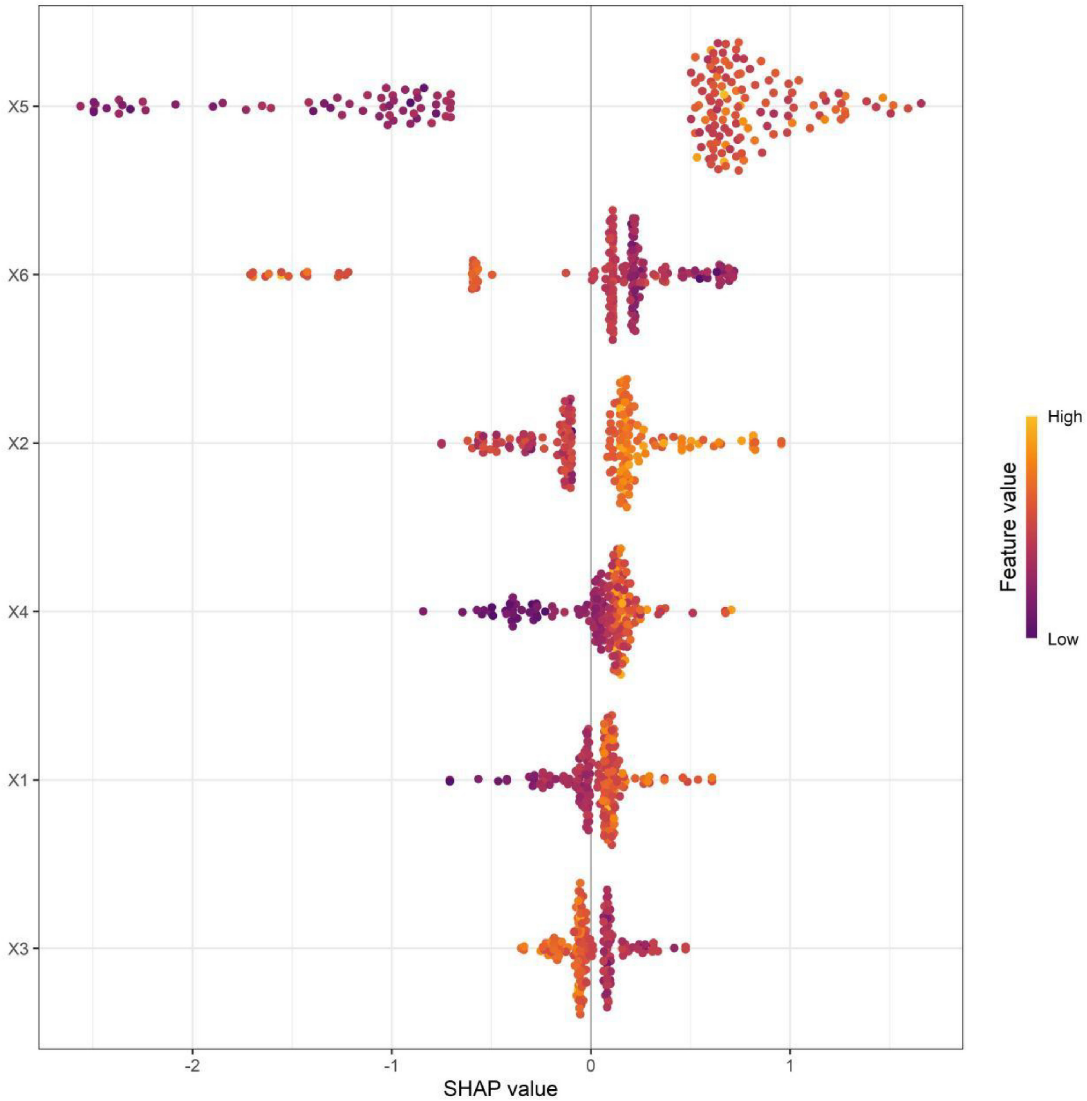
SHAP variable importance plot (X1, age; X2, vaginal pH value; X3, relative abundance of lactic acid bacteria; X4, HR-HPV viral load; X5, duration of persistent HPV infection; X6, serum vitamin D level).

Based on 6 core predictive features, a nomogram model was constructed to predict the occurrence of CIN in HR-HPV infected patients. Scores were assigned to each independent risk factor in the model, and the total score for predicting the occurrence of CIN in HR-HPV infected patients was calculated and presented as the predicted CIN probability. The higher the total score, the higher the risk of predicting the occurrence of CIN in HR-HPV infected patients (Figure 1). SHAP analysis further quantified the relative importance of each feature. In terms of the degree of influence, the duration of persistent HPV infection (X5), serum vitamin D level (X6), vaginal pH value (X2), HR-HPV viral load (X4), age (X1), and relative abundance of lactic acid bacteria (X3) successively showed an impact on the model prediction (Figure 2). Among them, the duration of persistent HPV infection, vaginal pH value, and HR-HPV viral load had a significant positive predictive contribution to the occurrence of cervical intraepithelial neoplasia; the serum vitamin D level and relative abundance of lactic acid bacteria had an obvious negative impact, indicating their protective effect. The results of SHAP analysis were consistent with the conclusions of multivariate Logistic regression, enhancing the clinical interpretability and rationality of the model.

### Evaluation and validation of the model

**FIGURE 3:**
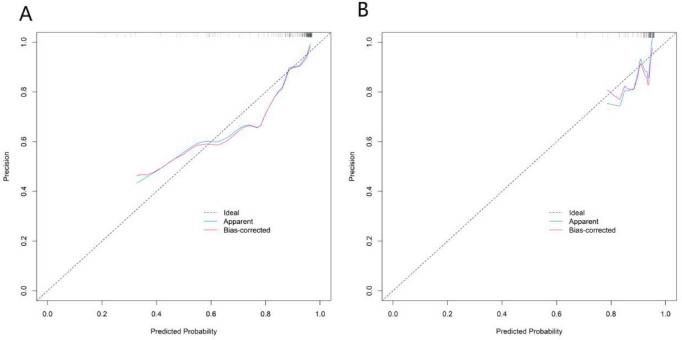
Calibration curves in the training set **(A)** and the validation set **(B)**.

**FIGURE 4:**
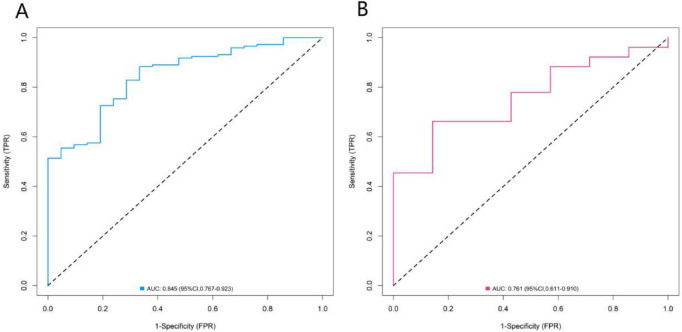
Receiver operating characteristic curves in the training set **(A)** and the validation set **(B)**.

In the training set and validation set, the C-indices of the nomogram model were 0.845 and 0.761, respectively. The calibration curves showed that the mean absolute errors between the predicted values and the actual values were 0.160 and 0.170, respectively. The results of the Hosmer–Lemeshow test showed that the χ^2^ values of the two groups of data were χ^2^ = 10.311 (*P* = 0.243) and χ^2^ = 6.208 (*P* = 0.624), respectively (Figure 3). The ROC curves showed that the AUC of the nomogram model for predicting acute exacerbation in the training set and validation set were 0.845 (95% CI: 0.767–0.923) and 0.761 (95% CI: 0.611–0.910), respectively. The sensitivities and specificities of the two groups of data were 0.660 and 0.845, and 0.619 and 0.725, respectively (Figure 4).

### Decision curve analysis of the model

**FIGURE 5:**
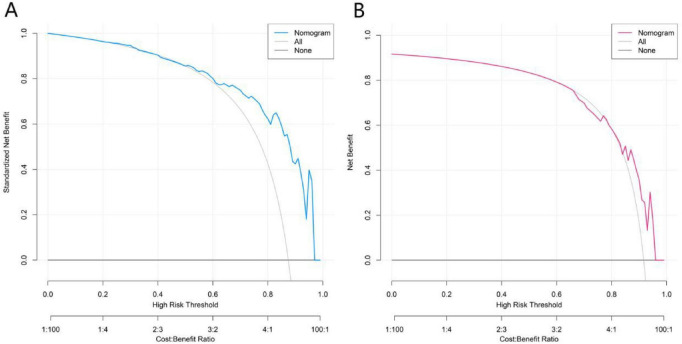
Decision curves in the training set **(A)** and the validation set **(B)**.

Decision curve analysis showed that in the training set model, within the high-risk threshold range of 0.0–0.9, the standardized net benefit of the Nomogram curve was significantly better than that of the extreme strategy, demonstrating the decision-making value in the training stage. The validation set model continuously maintained the net benefit advantage within the range of 0.0–0.9, and the applicable risk threshold range was wider. This indicated that the model not only had clinical decision-making value in the training stage but also had good generalization performance in the independent validation set. Especially in the higher-risk threshold range, it could still stably provide net benefits, reflecting reliable clinical practicability and promotion potential (Figure 5).

## Discussion

In this study, through multivariate Logistic regression analysis, age, vaginal pH value, relative abundance of lactobacilli, HR-HPV viral load, duration of persistent HPV infection, and serum vitamin D level were identified as independent influencing factors for the occurrence of CIN in patients with HR-HPV infection. Moreover, the weight and direction of the effect of each factor were verified by SHAP analysis, and its internal mechanism was highly consistent with clinical practice.

Age, as an independent risk factor, was consistent with the conclusions of previous studies. With increasing age, the physiological functions of the female reproductive system gradually decline. The proliferation and repair ability of cervical epithelial cells decreases, and the efficiency of clearing HR-HPV infection reduces. Meanwhile, the physiological decline of immune function weakens the body’s immune surveillance and defense against viral infections, thereby increasing the risk of atypical hyperplasia of epithelial cells (8, 9). In this study, the average age of the CIN occurrence group was significantly higher than that of the non-occurrence group. Multivariate analysis showed that for every 1-year increase in age, the risk of CIN occurrence increased by 17.5%. This suggests that age can serve as a simple indicator for the initial clinical screening of high-risk populations, providing a basis for the stratified management of HR-HPV infected patients of different age groups.

The synergistic effect of an increased vaginal pH value and a decreased relative abundance of lactobacilli further confirmed the crucial role of vaginal microecological imbalance in the occurrence of CIN. Under normal physiological conditions, lactobacilli in the vagina maintain an acidic environment with a pH value of 3.8–4.4 by producing lactic acid, inhibiting the proliferation of harmful microorganisms and forming a biological barrier against pathogen invasion (10). When the relative abundance of lactobacilli decreases, the acidic environment of the vagina is disrupted, and the pH value increases. This not only weakens the inhibitory effect on HR-HPV but may also promote viral adsorption, replication, and persistent infection by altering the local cervical microenvironment. At the same time, it reduces the defense ability of cervical epithelial cells and accelerates the occurrence and progression of atypical hyperplasia (11, 12). In this study, the vaginal pH value of the CIN occurrence group was significantly higher than that of the non-occurrence group, while the relative abundance of lactobacilli was significantly lower. Multivariate analysis showed that for every 1% increase in the relative abundance of lactobacilli, the risk of CIN occurrence decreased by 7.4%. This result provides direct experimental evidence for intervening in HR-HPV infection-related lesions by regulating the vaginal microecology (such as supplementing probiotics).

HR-HPV viral load and duration of persistent infection are the core risk factors for the occurrence of CIN, and their mechanism of action is closely related to the pathogenic characteristics of the virus. A high viral load means active replication of the virus in cervical epithelial cells, and the interference with cell cycle regulatory genes (such as p53 and Rb genes) is more significant, which easily leads to uncontrolled cell proliferation and abnormal differentiation (13). The persistent infection state provides the necessary conditions for the integration of the viral genome into the host cell chromosome, triggering the activation of proto-oncogenes and the inactivation of tumor-suppressor genes (14, 15). In this study, both the HR-HPV viral load and the duration of persistent infection in the CIN occurrence group were significantly higher than those in the non-occurrence group. For every 1 × 10^4^ copies/mL increase in viral load, the risk of CIN occurrence increased by 72.9%, and for every 1-month increase in the duration of persistent infection, the risk increased by 38.2%. This finding emphasizes the importance of monitoring the viral load and long-term follow-up of patients with HR-HPV infection, which can provide key references for judging the risk of lesion progression (16).

Serum vitamin D level, as a protective factor, may be related to its immunomodulatory function. Vitamin D activates the vitamin D receptor (VDR) to regulate the functions of immune cells (such as T cells, B cells, and macrophages), enhancing the body’s ability to clear viral infections. At the same time, it inhibits the over-activation of the inflammatory response and reduces oxidative stress damage to cervical epithelial cells (17, 18). In this study, the serum vitamin D level of the CIN occurrence group was significantly lower than that of the non-occurrence group. For every 1 ng/mL increase in vitamin D level, the risk of CIN occurrence decreased by 15.6%. This suggests that vitamin D deficiency may increase the risk of HR-HPV infection-related lesions by weakening immune defense and anti-inflammatory effects, providing theoretical support for clinical preventive intervention by supplementing vitamin D (19, 20).

The nomogram model constructed in this study integrates multi-dimensional indicators from clinical, microbiological, and serological perspectives, and exhibits prominent innovative advantages and clinical practical value. First, in terms of the prediction dimension, it breaks through the limitations of traditional single-factor assessment and realizes the comprehensive quantification of the risk of CIN occurrence. Previous studies mostly focused on the association between a single indicator (such as HPV typing and vaginal microecology) and CIN, failing to comprehensively consider the interaction of various factors. This model selects core indicators through multivariate Logistic regression, ensuring the comprehensiveness of prediction. Moreover, the relative importance of each indicator is clarified through SHAP analysis, making the prediction logic of the model clearer and more clinically interpretable.

Second, in terms of model performance, the model shows excellent discrimination and calibration. The C-index of the training set and the validation set reached 0.856 and 0.832, respectively, and the AUC was 0.863 and 0.845, respectively. Both the sensitivity and specificity were at a relatively high level, indicating that the model has good accuracy in predicting the risk of CIN occurrence. Meanwhile, the results of Bootstrap internal validation and external validation were consistent, confirming that the model has good stability and extrapolation ability and can be applied to HR-HPV infected patients in different clinical scenarios. In addition, the visual design of the nomogram transforms the complex statistical model into an intuitive scoring tool. Clinicians can quickly estimate the probability of CIN occurrence by simply inputting the patient’s various indicators without complex statistical calculations, greatly enhancing the clinical practicality of the model and providing a feasible tool for risk screening in primary medical institutions.

The results of decision curve analysis show that the model has good clinical net benefits within a wide range of threshold probabilities, indicating that the model can effectively identify high-risk populations, avoiding over-intervention and missed diagnosis. For high-risk patients, it is recommended to shorten the follow-up interval, strengthen monitoring, or take targeted intervention measures (such as vaginal microecological regulation and vitamin D supplementation). For low-risk patients, a routine follow-up strategy can be adopted to reduce unnecessary consumption of medical resources, achieving individualized and precise management of HR-HPV infected patients, which is in line with the development concept of modern precision medicine.

Nowadays, numerous studies have investigated the construction and clinical application of prediction models for HR-HPV infection-related CIN and cervical cancer. Huang et al. (21) enrolled 210 patients with HR-HPV infection as study subjects, screened safety precautions, serum progesterone level and the relative abundances of Lactobacillus acidophilus, Lactobacillus crispatus, Lactobacillus jensenii and Lactobacillus rhamnosus as independent risk factors, and established and validated a nomogram model for predicting CIN occurrence (21). This model showed high discrimination and calibration in both the training and validation cohorts. Koeneman et al. (22) developed and internally validated a model for predicting the spontaneous regression of cervical intraepithelial neoplasia grade 2 (CIN2) using simple clinical parameters including smoking, preoperative Pap smear results and concurrent CIN1 in biopsy as variables, providing a reference for individualized conservative treatment decisions for CIN2 (22). Zhou et al. (23) constructed a regression model for predicting cervical squamous cell carcinoma with hTERC gene amplification, HR-HPV viral load and MCM5 protein expression as core indicators (23). This model presented good fitting performance and a prediction accuracy as high as 98.5%. Our study included 358 patients with HRperformance and a prediction accuracy noma wittobacillus count, elevated vaginal pH, high HRnce and a prediction accuracy noma with hTERC gene amplification, HR-HPV viral load and MCM5 protein expreas independent risk factors for CIN occurrence, and further constructed a nomogram prediction model with favorable discrimination and calibration.

The advantages of this study are mainly reflected in the following aspects: First, the sample size was sufficient. A total of 358 patients were included strictly according to the sample size calculation results and divided into a training set and a validation set at a ratio of 7:3, meeting the statistical requirements for model construction and validation and ensuring the reliability of the results. Second, the outcome was clearly defined. Referring to the latest clinical guidelines and diagnostic criteria, through multi-time-point follow-up, double-blind review, and an arbitration mechanism, the accuracy of outcome determination was guaranteed. Third, multi-dimensional indicator detection and a standardized data collection process were adopted, combined with an EDC system for data management, reducing measurement bias and data loss and improving the research quality. Fourth, SHAP analysis was innovatively applied to model interpretation, quantifying the contribution and influence direction of each predictive factor and enhancing the clinical acceptability of the model.

Meanwhile, this study also has some limitations: First, it is a single-center retrospective study. All the included patients were from the same hospital, which may lead to selection bias and limit the extrapolation ability of the model to a certain extent. Follow-up research could conduct multi-center, prospective cohort studies to expand the coverage of sample sources, so as to further verify the extrapolation ability and clinical applicability of the model in different populations and clinical scenarios. econd, the indicators included in the model were all baseline data, without considering the dynamic changes of indicators during the follow-up (such as fluctuations in the abundance of lactobacilli and the intervention effect of vitamin D level), which may affect the prediction accuracy. Dynamic indicators such as lactobacillus abundance and serum vitamin D level can reflect the real-time changes of vaginal microecological regulation effect and the body’s immune status during follow-up, and their dynamic monitoring data can also reflect the progression trend of HR-HPV infection and cervical lesions. Incorporating such dynamic indicators into the prediction model is expected to enrich the prediction dimension, realize the dynamic assessment of CIN occurrence risk, and further improve the prediction accuracy and clinical application value of the model. In the future, a dynamic prediction model can be constructed, incorporating longitudinal data to improve the model performance Third, HPV genotyping, a key clinical indicator for HR-HPV infection and cervical lesion assessment, was not included in this study. Different HR-HPV subtypes have significant differences in pathogenicity, among which high-risk subtypes such as HPV16 and HPV18 have a much higher risk of inducing CIN and cervical cancer, and the study failed to analyze the differential impact of different subtypes on the risk of CIN occurrence. This deficiency leads to insufficient comprehensiveness of the model’s predictive indicators, and the model structure still has considerable room for optimization. In subsequent studies, the model can be further refined by incorporating HPV genotyping and analyzing the pathogenic differences of different subtypes to optimize the prediction effect and improve the clinical applicability of the model. Fourth, the interaction between the included predictive factors was not explored in depth, such as the potential synergistic or antagonistic effect between vaginal microecological indicators (lactobacillus abundance, vaginal pH) and serum vitamin D level on the risk of CIN occurrence. The lack of in-depth analysis of the interrelationship between factors makes it impossible to reveal the joint regulatory effect of multiple factors on the occurrence and development of CIN, which to a certain extent limits the depth of the study on the pathogenesis of CIN. In subsequent research, stratified analysis or interaction term testing can be used to explore the interaction between different predictive factors, so as to provide a more comprehensive theoretical basis for the mechanism of CIN occurrence and further optimize the prediction model.

In this study, a nomogram model for predicting the risk of CIN occurrence based on age, vaginal pH value, relative abundance of lactobacilli, HR-HPV viral load, duration of persistent HPV infection, and serum vitamin D level was successfully constructed and validated. This model has good discrimination, calibration, and clinical practicality and can provide individualized assessment of the risk of CIN occurrence for patients with HR-HPV infection.

The clinical application value of this model is mainly reflected in the following aspects: First, it provides a precise risk assessment tool for clinicians, which helps to identify high-risk populations early and formulate individualized follow-up and intervention strategies. Second, it provides clear targets for the preventive intervention of CIN, suggesting that the risk of lesions in high-risk patients can be reduced by regulating the vaginal microecology (supplementing lactobacilli) and supplementing vitamin D. Third, it provides a feasible solution for the screening of precancerous cervical lesions in primary medical institutions. Through simple and easily measurable indicators, risk stratification is achieved, and the screening efficiency is improved.

Future studies can further expand the sample size, conduct multi-center prospective studies to verify the extrapolation ability of the model; incorporate more potential predictive factors (such as HPV typing and immune function indicators) to optimize the model structure; construct a dynamic prediction model, combining the changes of indicators during the follow-up to improve the prediction accuracy; at the same time, conduct interventional studies based on the model to verify the effect of targeted intervention measures on reducing the risk of CIN occurrence, providing more comprehensive evidence-based medical evidence for the clinical management of HR-HPV infected patients, ultimately reducing the incidence of cervical cancer and safeguarding female reproductive health.

## Data Availability

The original contributions presented in this study are included in the article/supplementary material, further inquiries can be directed to the corresponding author.
